# Burden of neck pain among medical students in Ethiopia

**DOI:** 10.1186/s12891-019-3018-x

**Published:** 2020-01-08

**Authors:** Gidey Gomera Weleslassie, Hagazi Gebre Meles, Tsiwaye Gebreyesus Haile, Gebreslassie Kahsay Hagos

**Affiliations:** 1 Department of Physiotherapy, School of Medicine, College of Health Sciences and Ayder Comprehensive Specialized Hospital, Mekelle University, Mekelle, Tigray Ethiopia; 2 Department of Biostatistics, School of Public Health, College of Health Sciences and Ayder Comprehensive Specialized Hospital, Mekelle University, Mekelle, Tigray Ethiopia

**Keywords:** Neck pain, Medical students, Associated factors, Standard Nordic questionnaires

## Abstract

**Background:**

Neck pain is the major cause of morbidity and absenteeism from university lessons among medical students worldwide. Medical students are more exposed and appear to have neck pain because of their length of study to achieve their professional goals. However, up to the knowledge of the researcher, there is a scarcity of literature conducted on prevalence and associated factors of neck pain among medical students in Ethiopia. Therefore, the aim of this study was to determine the prevalence and factors associated with neck pain among medical students at Mekelle University, College of Health Sciences, Tigray, Ethiopia.

**Method:**

Institutional based cross-sectional study was conducted from April 2018 to May 2018. A structured questionnaire adapted from the Nordic musculoskeletal questionnaire was distributed to 422 participants using a self-administered questionnaire in Mekelle University, College of Health Sciences Tigray, Ethiopia. Independent variables which had a significant association were identified using logistic regression models. Results were reported by using texts and frequency distribution tables.

**Result:**

A total of 422 participants involved in this study, with a 99.3% response rate. Previous 12 months self-reported prevalence of neck pain among medical students was found to be 49.2% with 95%CI (44.5–54%). Previous history of neck pain (AOR: 11.811, 95%CI: 5.460–25.549), physical exercise (AOR: 2.044, 95%CI: 1.233–3.387), duration of reading (AOR: 1.502, 95%CI: 0.236–2.780) and awkward posture (AOR: 3.87, 95%CI: 2.311–6.484) were factors significantly associated with neck pain.

**Conclusion and recommendation:**

The current study showed that nearly half of the study participants self-reported to have suffered neck pain in the preceding 12 months. Past history of neck pain, physical exercise, duration of reading and awkward neck posture are likely to be significantly associated neck pain among medical students in Ethiopia. Engaging in consistent physical exercise has a protective effect against neck pain. Therefore, Medical students are recommended to carry out a regular physical exercise for a minimum of twenty to thirty minutes per day.

## Background

Neck pain (NP) is increasingly becoming a health problem and has a considerable socio-economic impact on individuals, their families and communities [1, 2]. Neck pain is a major cause of sickness, reduced educational attainment and truancy from university lessons which will affect students’ future careers [1].

The principal aims of a medical school are to produce capable, professional doctors and promote health care of society. But during the period of medical training, they expose students to stress, study problems, long training hours in hospital wards and clinics during the period of their medical training [3]. Medical students seemed to have a higher risk of developing NP compared to the general population [4]. Besides the factors predisposing to pain in the general population, students subject themselves to hours of prolonged reading, writing, and being in the clinical practice which makes them high-risk group for NP. Furthermore, computer or tablet use is very common among medical students [5].

Previous studies have reported a high prevalence of NP in medical students. A study conducted at a Malaysian medical college found that 41.8% of students had NP within the past year and reported an association with clinical years, computer use and a prior history of trauma [3]. Studies conducted at Pakistan, Australia, New York, United States of America (USA), China and Brazil found that 65, 52.8, 35, 33.8, 8.23% of medical students had NP respectively [6–10]. Another study at Central Saudi Arabia reported a prevalence of 56.5% for NP among medical students [11] and a study in Nigeria revealed that the lifetime prevalence of NP among the respondents was 34.9% [12].

Neck pain has a multi-factorial origin, and there are several factors contributing to its onset and perpetuation. According to Guzman and his colleagues, physical, psychosocial and individual-related factors were the most reportable factors of NP among medical students [13]. It can be caused by interference surrounding anatomical neck structures like nerve, airway, vascular, musculoskeletal, prolonged activity, poor posture and history of previous neck injury [14].

Socio-demographic factors for example gender, age, body mass index (BMI), behavioral and psycho- social factors like smoking, drinking habit, physical exercise, stress, sleeping hours, physical factors, for instance, tablet or computer use, long sitting hours, seats without back supports, duration of reading, repetitive movements and awkward posture are considered as associated factors of NP among medical students [3, 6, 8, 10–12, 15–26]. Because of this in explicit pain, medical students undergo frequent sick leave, medical disability, functional impairments, decreased productivity, health cost and absenteeism from university lessons [1, 27]. Our extensive search shows a scarce of published regional reports the prevalence of NP among medical students in the sub-Saharan region, and they found none in Ethiopia.

The purpose of this study was to assess the prevalence and identify factors associated with NP among medical students in Mekelle University (MU), College of Health Sciences (CHS), Tigray, Ethiopia.

## Methods

### Study design, period and study area

An institutional based cross-sectional study was conducted from April to May 2018 at Mekelle University, College of Health Sciences, Tigray, Ethiopia.

Mekelle, the capital city of Tigray region is found 780 km away to North direction from Addis Ababa, the capital city of Ethiopia. Mekelle University (MU) has seven colleges and five campuses, College of Health Sciences (CHS) is one of them, has about 4000 students. Out of them 1308 are undergraduate medical students that become medical doctors. It is one of the largest undergraduate medical training institutions in Northern Ethiopia (http://www.mu.edu.et/chs/index.php/ayderreferral-hospital). The medical school curriculum involves basic science in pathology and disease with early clinical exposure during the first year to the third year, required clinical rotations, elective rotations and internship during the fourth year to seventh years.

### Source and study population

All undergraduate medical students at MU, CHS were used as a source population and all randomly selected medical students from the first to sixth years of medical study in MU, CHS were study subjects.

### Inclusion criteria

All undergraduate medical students at MU, CHS who were available at the time of the data collection were eligible to participate.

### Exclusion criteria

Students with a well-known health problem (recent trauma or surgery) around the neck area. We prepared a checklist with “Yes” or “No” questions prior to data collection to check whether the participants had a known health problem and participants who responded “Yes” were excluded from this study. The final year students were excluded because they had already graduated from the university when our study began.

### Sample size and sampling methods

The sample size of the study was calculated using a single population proportion formula by considering 50% prevalence of NP, 95% confidence interval and 5% margin of error [28].

$$ {\displaystyle \begin{array}{c}\mathrm{N}=\frac{{\left({z}_{\alpha }/2\right)}^2\mathrm{P}\left(1-\mathrm{P}\right)}{{\mathrm{D}}^2}\\ {}\mathrm{N}=\frac{(1.96)^20.5\left(1-0.5\right)}{(0.05)^2}=384\end{array}} $$

Where; *P* = 50% proportion prevalence of NP.

Zα/2 = critical value of the Z score at a 95% confidence interval.

D = margin of error (5% (0.05).

Finally, the sample size of 422 was obtained by addition of a 10% non-response rate.

**Fig. 1:**
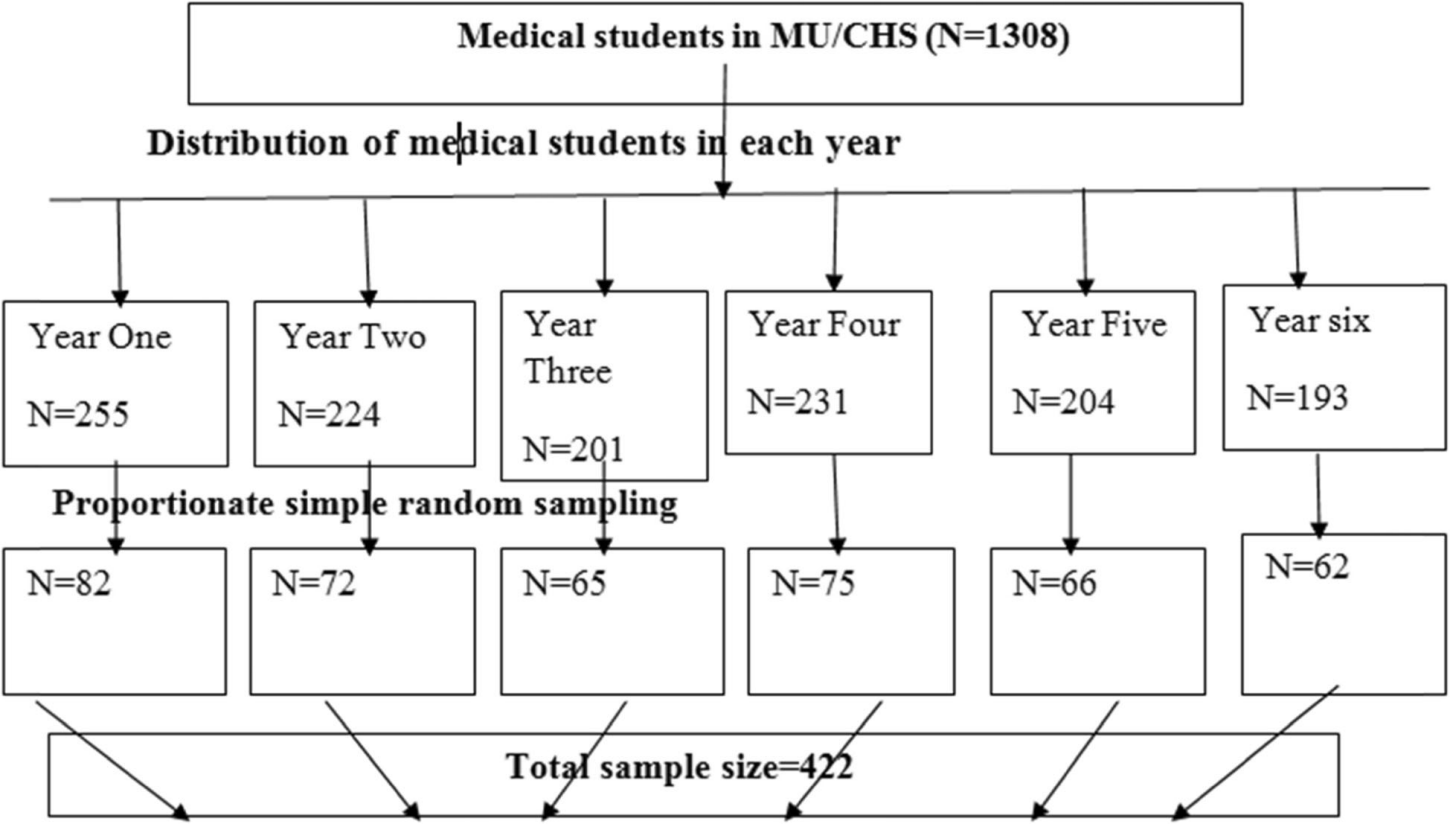
Schematic representation of sampling procedure

The students were distributed in to six based on their year of study (batches). From each year the samples were proportionally allocated based on the total number of students. Finally, a lottery method was recruited using their list from the registrar and alumni to select the actual participants (See Fig. 1).

### Data collection procedures and quality control

Data was collected using structured self-administered questionnaires. A tool used to assess NP was adapted from the standardized Nordic questionnaire [29] and it was modified to the local context. Based on this questionnaire, NP for the last 12 months was asked by: “Have you at any time during the last 12 months had trouble (ache, pain or discomfort?) in your neck”. The questionnaire was used as an instrument for gathering NP and it was available in the English language. The perceived stress scale (PSS)-10 was used to assess the degree of self-perceived stress of medical students [30]. The questionnaire was composed of three sections. Section1, included questions on socio-demographic data. Section 2, behavioral and psycho-social factors such as a history of cigarette smoking, alcohol consumption, a habit of physical exercise and self-perceived stress. Section 3, physical factors such as computer /laptop uses, duration of reading, static head down posture, awkward posture, comfortable back support and the occurrence of NP in the last 12 months. The questionnaire was pre-tested at Adigrat University away from the study area on twenty-one students before distributing among the participants to ensure the understandability of the questions. The questionnaires were distributed separately to each of the first to six batches at the end of their classes. Data collection was done by four trained physiotherapists to assess NP. The principal investigator and the supervisors throughout the data collection period closely monitored the data collection process. Filled questionnaires were checked daily for the completeness of information and conflicts were reported to data collectors.

### Operational definition

#### Neck pain

NP was defined as pain, ache or discomfort in the area between the occiput and the first thoracic vertebra at any time in the last 12 months [31].

### Data management and analysis

Data were coded and entered into Epi info software version 7.0 and analyzed using the IBM Statistical Package for Social Sciences (SPSS) version 23 for Windows [32]. The results were presented using text, frequency distribution tables and percentages for descriptive statistics. Binary logistic regression was used to identify factors associated with NP. Bivariate analysis was done to see the association between NP and independent variables. Variables with a *P*-value less than 0.25 were brought to multivariate analysis for controlling potential confounding factors. The model fitness was checked using log-likelihood, Hosmer and Lemeshow goodness fit test. Multicollinearity test was also checked with a variance inflation factor (VIF) > 2.5 were considered significant to assess the correlation between the independent variables. The assumptions of each categorical variable having five and more than five cells were considered. Independent variables with a 95% confidence level and *P*-value less than 0.05 in the multivariate model was considered as statistically significant and presented with Adjusted Odds Ratio (AOR) with 95% CI.

## Results

### Socio-demographic characteristics of medical students

A total of 422 questionnaires were distributed, of which 419 students responded, hence the response rate was 99.3%. The majority of the study participants (65.6%) were males. The respondents mean age was 22 years (SD ± 2.215 years). The majority of the study participants (64.0%) were in the age group of 21–25 years and (97.9%) were single. More than half of the participants 216 (51.6%) were in the pre-clinical year. Preponderantly BMI in the participant’s 336 (80.2%) was categorized as normal weight and followed by underweight 64 (15.3%).

A minority of the respondents (20.0%) were having a history of NP (Table 1).

**Table 1:** Socio-demographic characteristics of medical students at MU, CHS, April, 2018 (*N* = 419)

Variables	Frequency (N)	Percent (%)
Sex		
Male	275	65.6%
Female	144	34.4%
Age		
≤ 20	137	32.7%
21–25	268	64.0%
≥ 26	14	3.3%
Religion		
Orthodox	350	83.5%
Protestant	26	6.2%
Muslim	33	7.9%
Other	10	2.4%
Marital status		
Single	410	97.9%
Married	9	2.1%
Year of study		
Preclinical	216	51.6%
Clinical	203	48.4%
BMI		
Underweight	64	15.3%
Normal	336	80.2%
Overweight	19	4.5%
Past history of NP		
Yes	84	20.0%
No	335	80.0%

### Behavioral and psychosocial characteristics

Merely 3.3% of students were smokers and 27.9% were alcohol drinkers. A minority of the respondents (35.1%) were involved in regular physical exercises for more than 150 min per week.

The majority of the participants 342 (81.6%) were having moderate stress, 56 (13.4%) low stress, and 240 (57.3%) seven to eight sleeping hours duration (Table 2).

**Table 2:** Behavioral and psychosocial characteristics of medical students at MU, CHS, April, 2018 (*N* = 419)

Variables	Frequency (N)	Percent (%)
Physical exercise		
Yes	147	35.1%
No	272	64.9%
Cigarette smoking		
Yes	14	3.3%
No	405	96.7%
Alcohol drinking		
Yes	117	27.9%
No	302	72.1%
Self-perceived stress		
Low stress	56	13.4%
Moderate stress	342	81.6%
High stress	21	5.0%
Sleeping hours		
≤ 6 h	127	30.3%
7–8 h	240	57.3%
≥ 9 h	52	12.4%

### Physical characteristics

The majority of medical students, 400 (95.5%) use a computer or tablet and 234 (55.8%) of them do not use a comfortable back support.

More than half of participants, 214 (51.1%) that they were using a static head down posture and164 (39.1%) of the participants with awkward neck posture for 2 h a day. The majority of respondents, 366 (87.4%) were seated for a long period (Table 3).

**Table 3:** Physical characteristics of NP among medical students at MU, CHS, April, 2018 (*N* = 419)

Variables	Frequency (N)	Percent (%)
Laptop/tablet use		
Yes	400	95.5%
No	19	4.5%
Hours of laptop/tablet use		
< 2 h/day	46	11.5%
≥ 2 h/day	354	88.5%
Prolong Sitting (> 2 h)		
Yes	366	87.4%
No	53	12.6%
Duration of study/reading		
< 3 h/day	76	18.1%
≥ 3 h/day	343	81.9%
Static head down posture		
Yes	214	51.1%
No	205	48.9%
Awkward neck posture		
Yes	164	39.1%
No	255	60.9%
Repetitive neck movement		
Yes	137	32.7%
No	282	67.3%
Back support		
Yes	185	44.2%
No	234	55.8%

### Prevalence of neck pain among medical students

The overall previous 12 months self-reported prevalence of NP among medical students was 49.2% (95% CI: 44.5–54%).

The prevalence of NP was higher among male students, students who have a past history of NP, students who have high stress, students who used laptop or tablet for two or more hours, medical students those who were using a static head down posture for more than 2 h during a lecture 52.0, 88.2, 61.9, 50.6 and 57.5% respectively. The prevalence of NP was nearly the same among the pre-clinical year (48.6%) and a clinical year (49.8%) medical students.

The prevalence of NP was less among those who had practiced a physical exercise (42.9%) and medical students those who did not use comfortable back support reported higher (52.1%) prevalence of NP.

### Factors associated with NP among medical students

In the bivariate logistic regression analysis, self-reported of NP was significantly associated with age, sex, cigarette smoking, alcohol drinking, regular physical exercise, history of NP, Stress, computer /laptop use, duration of reading, static head down posture, awkward neck posture, repetitive neck movement, prolonged sitting and back support. However, in the multivariate logistic regression analysis, self-reported of NP was associated significantly (*p* < 0.05) with regular physical exercise (AOR = 2.044, 95% CI: 1.233–3.387), past history of NP (AOR: 11.811, 95% CI: 5.460–25.549), duration of reading (AOR = 1.502, 95% CI: 0.236–2.780) and awkward neck posture (AOR: 3.87, 95%CI: 2.311–6.484) (Table 4).

**Table 4:** Bivariate and multivariate logistic regression analysis of associated factors with NP among medical students

Variables	Neck pain		COR (95% CI)	*P*-value	AOR (95% CI)	*P*-value
	Yes	No				
Sex						
Male	143 (52.0%)	132 (48.0%)	1.00			
Female	63 (43.8%)	81 (56.3%)	0.718 (0.479–1.077)	0.109		
Age						
≤ 20	62 (45.3%)	75 (54.7%)	1.00			
21–25	133 (49.6%)	135 (50.4%)	1.192 (0.789–1.801)	0.405		
≥ 26	11(78.6%)	3 (21.4%)	4.435 (1.185–16.607)*	0.027		
Cigarette smoking						
Yes	11 (78.6%)	3 (21.4%)	3.949(1.085–14.365)*	0.037		
No	195 (48.1%)	210 (51.9%)	1.00			
Alcohol drinking						
Yes	74 (63.2%)	43 (36.8%)	2.216 (1.428–3.439)*	< 0.001		
No	132 (43.7%)	170 (56.3%)	1.00			
Physical exercise						
Yes	63 (42.9%)	84 (57.1%)	1.00		1.00	
No	143 (52.6%)	129 (47.4%)	**1.478 (0.987–2.214)**	0.050	2.044 (1.233–3.387)**	0.006*
Past history of NP						
Yes	74(88.2%)	10 (11.8%)	11.380(5.675–22.820)*	< 0.001	11.811(5.460–25.549)**	< 0.001*
No	132(39.4%)	203 (60.6%)	1.00		1.00	
Self-perceived stress						
Low	23 (41.1%)	33 (58.9%)	1.00			
Moderate	170 (49.7%)	172 (50.3%)	1.418 (0.800–6.515)	0.232		
High	13 (61.9%)	8 (38.1%)	2.332 (0.833–6.525)	0.107		
Tablet/laptop use						
Yes	200 (50.0%)	200 (50.0%)	2.167 (0.808–5.813)	0.125		
No	6 (31.6%)	13 (68.4%)	1.00			
Duration of reading						
< 3 h	32 (42.1%)	44 (57.9%)	1.00		1.00	
≥ 3 h	174 (50.7%)	169 (49.3%)	1.416 (0.857–2.339)	0.175	1.502 (0.236–2.780)**	0.031*
Static head down posture (> 2 h)						
Yes	123 (57.5%)	91(42.5%)	1.987(1.347–2.931)*	0.001		
No	83 (40.5%)	122 (59.5%)	1.00			
Awkward neck posture						
Yes	108 (65.9%)	56 (34.1%)	3.089 (2.051–4.654)*	< 0.001	3.871(2.311–6.484)**	< 0.001*
No	98 (38.4%)	157 (61.6%)	1.00		1.00	
Repetitive neck movement						
Yes	83 (60.6%)	54 (39.4%)	1.987 (1.311–3.011)*	0.001		
No	123 (43.6%)	159 (56.4%)	1.00			
Prolong sitting (> 2 h)						
Yes	186 (50.8%)	180 (49.2%)	1.705 (0.943–3.082)	0.077		
No	20 (37.7%)	33 (62.3%)	1.00			
Back support						
Yes	84 (45.4%)	101 (54.6%)	0.764 (0.890–1.928)	0.171		
No	122 (52.1%)	112 (47.9%)	1.00			

* = significant association (bivariate), ** = significant association (multivariate), COR = crude odds ratio, AOR = adjusted odds ratio, 1.00 = references, * = *p*-value < 0.05

## Discussion

This is the primary study that investigated the prevalence of NP and the association of individual, psycho social and physical characteristics among medical students NP. The previous twelve-month self-reported prevalence of NP among medical students was 49.2% (95% CI: 44.5–54%), the results indicated that NP is a common health problem among medical students. This finding is consistent with similar studies conducted in Pakistan (44.8%) [26], Australia (52.8%) [8] and Central Saudi Arabia (54%) [33]. However, the prevalence of this study is higher than the studies conducted in Brazil (8.23%) [10], Thailand (22.3%) [34], China (33.8%) [9], United States of America (35%) [6], Iran (39.4%) [35], Malaysia (41.8%) [3] and Nigeria (34.9%) [12].

This difference observed in the prevalence rate of NP could be due to the differences in the study area, sample size, sampling method, and assessment tools. The study in Brazil has used convenience sampling technique with a small sample size [10] and the study from Iran was used the Oswestry questionnaire and descriptive analytical study design with a small sample size [35].

The study of China was used different methods of assessment of NP for medical students and used survey among all fourth-year students with a small sample size [9] and study from Nigeria used a mixed population of medical and non-medical students [12].

Studies from the USA and Malaysia used a survey and an online self-administered questionnaire. Besides, it could be because of high awareness regarding NP among the USA and Malaysian medical students [3, 6].

The study of Thailand [34] has used a cohort study with small sample size. But the present study has used a cross-sectional study and simple random sampling technique with fairly large sample size, and it includes only medical students from the year one -year six. The other reasons could be a facility provided for the students at their institution or social and economic differences between Ethiopia and the countries of the studies mentioned, the way in which classes, the library, and clinical places were organized and the protective factors involved contribute to the differences observed compared to the present study. In addition to this, psychosocial, and physical characteristics may increase the prevalence rate of NP among Ethiopian medical students. Furthermore, this study’s prevalence was lower than studies conducted in Nevada, Las Vegas (84.6%) [36], Pakistan (65%) [7], and another study from the USA (56%) [37]. The possible reasons could be due to a different sampling technique, data collection procedure and sample size. Studies conducted in Nevada, Las Vegas, and the USA included students by purposive sampling and mailing for data collection. The study done in Pakistan used non-probability convenience sampling technique, a mixed population (medical and non-medical students) with small sample size and interview type of data collection procedure [7]. But this study used simple random sampling technique, a large sample size with a self-administered type of data collection procedure.

In this study, the factors associated with NP were past history of NP, a physical exercise, duration of reading and bending or twisting in an awkward neck position.

This study found that medical students who had a past history of NP symptoms were approximately12 times more likely to develop NP as compared to those without past history of NP. This result was consistent with studies have done in Central Saudi Arabia and Malaysia [3, 33]. The significant association between NP and past history of NP in the present study is understandable, and the possible explanation is because of those individuals who had a previous history of NP symptoms were at a higher risk developing NP. Beside, persistent NP can have broad and profound effects on wellbeing with significant impairment of physical and psychological health [34].

This study showed that medical students who were not doing a regular physical exercise were 2 times more likely to develop NP than those who were doing a regular physical exercise. This result was consistent with the studies have done in the USA [22]. The possible explanation could be shortened and weak muscles can cause NP as they can cause misalignment of neck anatomical structures. In contrast, medical students those who did a regular physical exercise can strengthen, lengthen, improve flexibility and make their muscles and ligaments strong to support and keep the neck alignment for proper functioning and preventing injury [38]. A prior study in America showed those who participated in sports activities were less likely affected by musculoskeletal pain of the upper body [7].

Experiencing a twisting or bending position during a computer or tablet use and a clinical attachment has resulted in 3.87 times higher odds of NP in this study. Studies from the United States of America, Brazil, and Thailand, medical students reported a similar association [6, 10, 16]. This association could be explained as an awkward position, including excessive flexion and rotation of the neck, increases muscle tension and resulted in spasm and musculoskeletal symptoms [39]. On the other hand, awkward posture was not significantly consistent with the musculoskeletal study conducted in New York, United States of America [6]. This could be due to a high awareness of body mechanics or ergonomics factors, especially physical factors among USA medical students [40].

Medical students who read three or more hours per day with a static head down posture were 1.5 times more likely to develop NP compared to those who read for less than 3 h per day. This result was similar to studies done in Pakistan and Thailand [26, 34]. The possible reasons could be due to clinical exposure, overload of exams for long duration to perform sustained and frequent activities such as reading, clinical procedures inwards and other during their study year repeatedly under unfavorable positions could, lead to discomfort, muscle stiffness or tightness around the cervical region, eventually lead to NP [41].

Another study in Uttar found that the most common cause of NP among medical students was prolonged reading followed by the use of computers and prolonged writing [42]. Prolonged sitting during studying, positions assumed during lectures are related to a high prevalence of NP [34]. Also, long study hours were found to be greater among medical undergraduates but no significant association was found in medical students [7].

### Limitation of the study

Considering the remunerations of future research, there are a few limitations to be stated. This study was carried out in just one institute, ergonomic evaluation of a posture of students while using a computer or during the study was not done. The cross-sectional nature does not allow inferring of causality and effect. Another limitation of this study was the possibility of recall bias and the impact of a 12 month recall question. Since it was self-reported which could lead to over or under-estimation of the true prevalence and height and weight of respondents were self-reported.

## Conclusion

The findings of this study showed that NP is a common health problem among medical students in Ethiopia. Nearly 50 % of the study participants self-reported to have suffered NP in the preceding 12 months. Socio-demographic or individual related characteristics like past history of NP and lack of physical exercise, physical characteristics such as awkward neck posture and duration of reading were associated with NP. The medical school authorities are recommended to provide facilities to enhance physical activity among medical students and students are recommended to develop an awareness of related health hazards and encourage the habit of regular physical exercise.

## Data Availability

Additional data could be obtained from the corresponding author upon formal request.

## Abbreviations

AOR: Adjusted odds ratio
BMI: Body mass index
CHS: College of health sciences
COR: Crude odds ration
MU: Mekelle University
NP: Neck pain
PSS: Perceived stress scale
SD: Standard deviation
USA: United States of America
